# The contribution of G-layer glucose in *Salix* clones for biofuels: comparative enzymatic and HPLC analysis of stem cross sections

**DOI:** 10.1186/s13068-022-02123-z

**Published:** 2022-03-05

**Authors:** Jie Gao, Mohamed Jebrane, Nasko Terziev, Geoffrey Daniel

**Affiliations:** Department of Forest Biomaterials and Technology/Wood Science, Swedish University of Agricultural Sciences, Box 7008, SE–750 07 Uppsala, Sweden

**Keywords:** *Salix viminalis*, Tension wood quantification, Gelatinous layer, Stem sections, Enzymatic and HPLC profiling, Biofuels

## Abstract

**Background:**

Interest on the use of short rotation willow as a lignocellulose resource for liquid transport fuels has increased greatly over the last 10 years. Investigations have shown the advantages and potential of using *Salix* spp. for such fuels but have also emphasized the wide variations existing in the compositional structure between different species and genotypes in addition to their effects on overall yield. The present work studied the importance of tension wood (TW) as a readily available source of glucose in 2-year-old stems of four *Salix* clones (Tora, Björn, Jorr, Loden). Studies involved application of a novel approach whereby TW-glucose and residual sugars and lignin were quantified using stem cross sections with results correlated with HPLC analyses of milled wood. Compositional analyses were made for four points along stems and glucose derived from enzyme saccharification of TW gelatinous (G) layers (G-glucose), structural cell wall glucose (CW-glucose) remaining after saccharification and total glucose (T-glucose) determined both theoretically and from HPLC analyses. Comparisons were also made between presence of other characteristic sugars as well as acid-soluble and -insoluble lignin.

**Results:**

Preliminary studies showed good agreement between using stem serial sections and milled powder from *Salix* stems for determining total sugar and lignin. Therefore, sections were used throughout the work. HPLC determination of T-glucose in *Salix* clones varied between 47.1 and 52.8%, showing a trend for higher T-glucose with increasing height (Björn, Tora and Jorr). Using histochemical/microscopy and image analysis, Tora (24.2%) and Björn (28.2%) showed greater volumes of % TW than Jorr (15.5%) and Loden (14.0%). Total G-glucose with enzyme saccharification of TW G-layers varied between 3.7 and 14.7% increasing as the total TW volume increased. CW-glucose measured after enzyme saccharification showed mean values of 41.9–49.1%. Total lignin between and within clones showed small differences with mean variations of 22.4–22.8% before and 22.4–24.3% after enzyme saccharification. Calculated theoretical and quantified values for CW-glucose at different heights for clones were similar with strong correlation: T-glucose = G-glucose + CW-glucose. Pearson’s correlation displayed a strong and positive correlation between T-glucose and G-glucose, % TW and stem height, and between G-glucose with % TW and stem height.

**Conclusions:**

The use of stem cross sections to estimate TW together with enzyme saccharification represents a viable approach for determining freely available G-glucose from TW allowing comparisons between *Salix* clones. Using stem sections provides for discrete morphological/compositional tissue comparisons between clones with results consistent with traditional wet chemical analysis approaches where entire stems are milled and analyzed. The four clones showed variable TW and presence of total % G-glucose in the order Björn > Tora > Jorr > Loden. Calculated in terms of 1 m^3^, *Salix* stems Tora and Björn would contain ca. 0.24 and 0.28 m^3^ of tension wood representing a significant amount of freely available glucose.

**Graphical Abstract:**

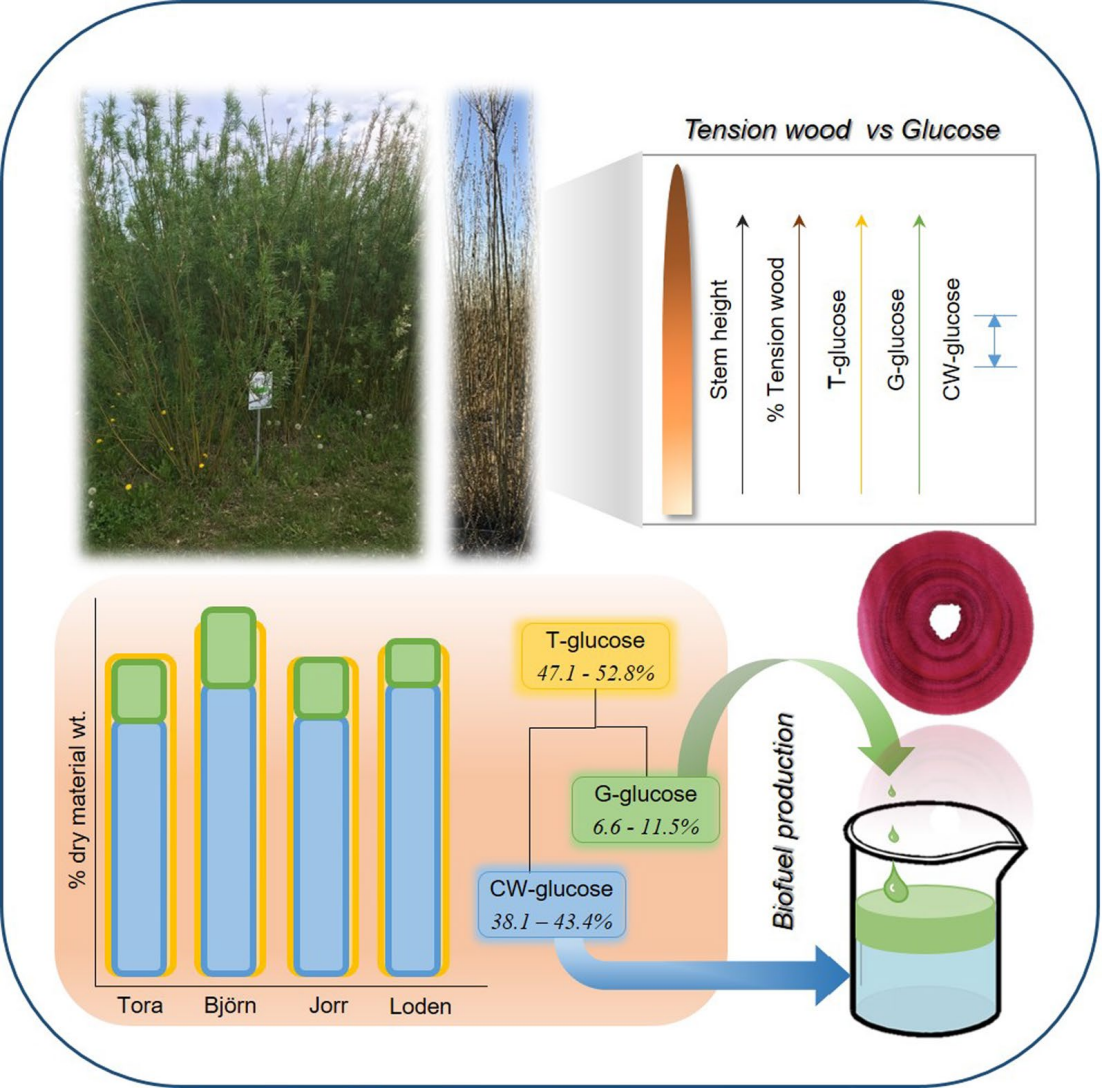

## Background

Recent changes in society have spurned greater interest in the use of biomass for biofuels and biogas [1–4]. Processing biomass for liquid transport fuels involves many steps of which one of the most important is choice of the native biomass and the process technology available [5–7]. Biomass is composed of three major polymers (cellulose, lignin, hemicelluloses) that can vary considerably according to plant species. In plant cell walls, these polymers are normally distributed at the molecular level with cellulose embedded in a “hemicellulose-lignin” matrix [8]. This represents the supramolecular basis of plant cell wall recalcitrance and while variations occur at both tissue and cell wall level in polymer microdistribution, it is the fundamental characteristic controlling hydrolysis of the plant secondary wall polymeric structure [9–11]. Thus, in order to release polymers from plant cell walls, biomass is normally pre-treated and broken down using physical or chemical-physical approaches to smaller entities with larger surface area to volume to increase access to the sugars and thus improve the hydrolysis potential [9–11]. A complementary approach is use of biomass with relatively low lignin content (e.g., fast growing plant species like *Salix,* Poplar) and here genetic approaches, including modification of lignin type and level, are of interest [12–19], i.e., to manufacture plant cell walls that break down more easily through greater access to the main sugar polymers of interest [13]. Of the three main plant polymers, the most interesting for biofuels is cellulose composed of β-1–4-linked glucose units. Rapid growing plant species (i.e., *Salix*) with high cellulose and low lignin content (especially of the syringyl type) are therefore most attractive for processing to biofuels. The cellulose content of hardwoods, like *Salix* spp., is generally reported as 40–50% [20, 21] although the amount of cellulose in fast growing *Salix* spp. has been variously reported as ranging from ca. 35–42% [22] to ca. 56% [23]. However, different methods of analysis have been used, including wet chemistry (e.g., Szczukowski et al. [23]), High-resolution thermogravimetric analysis (HR-TGA) (e.g., Serapiglia et al. [17]), and Near Infrared Spectroscopy (NIR) [24, 25]. Since some of the above analyses have been carried out on the same *Salix* spp., there is a strong possibility therefore that both natural variations in chemical composition and variations in methodology exist [26–28]. While local growing conditions can also affect plant development, an alternative explanation may be that part of the variation in total *Salix* biomass cellulose is derived from the variable presence of reaction wood known as tension wood (TW) in addition to native cell wall structural cellulose [27].

Tension wood is a characteristic tissue type that hardwoods develop in order to maintain branches in a more perpendicular orientation against the main tree trunk [29]. Tension wood also develops in rapid growing plants, like *Salix,* where its position in the stem axis is variably uni- or multilateral [30]. The most interesting characteristic of tension wood, however, is the development of gelatinous fibers (G-fibers) characterized by the synthesis of an essentially “lignin-free” inner cell wall layer called the gelatinous layer (G-layer) [29, 31, 32]. Thus, plant species with high cellulose and abundant tension wood should have great potential for biofuels that would be at least partly independent of the presence of lignin. For example, recent studies by Ray et al. [22] of 35 *Salix* genotypes showed wide variation in glucan yields in untreated and following acid and enzymatic hydrolysis that was independent of lignin content, a result that would be consistent with a variable presence of easily available glucose from G-layers. The problem, however, is to measure the G-fiber trait quantitatively allowing comparison between interesting clones. In a previous study [33], we developed an enzymatic method using *Salix* stem cross sections to quantify the amount of readily available enzymatic cellulose (i.e., glucose) in the gelatinous layer (i.e., G-glucose) of tension wood fibers in stems of *Salix*. The method was developed to quantify TW in *Salix* stems to allow comparisons between different clones and changes along the stem axis. In our previous work, quantitative determination of G-glucose was compared with an average value (45%) of total cellulose taken for *Salix* spp. from the literature using wet chemistry (i.e., HPLC analyses) [30, 33]. In the present study, we have further refined our approach using four 2-year-old commercial *Salix* clones (Tora, Björn, Jorr, Loden), to allow comparisons between (i) Glucose derived from the G-layer from TW fibers in stem sections (i.e., G-glucose from enzyme saccharification) with (ii) Cell wall glucose (i.e., CW-glucose remaining in stem cross sections after enzyme saccharification) and (iii) Total stem glucose (i.e., G-glucose + CW-glucose). Comparisons of glucose are made at four stem heights and related to the percentage area of TW present. In this way, it was possible to quantify and determine the contribution and importance played by G-layer glucose to the total glucose (cellulose) in *Salix* biomass and thus distinguish it from that derived from the structural cell wall glucose.

## Results and discussion

### Comparison between using stem cross sections and powder for determining the chemical composition of *Salix* wood samples

Most commonly, entire plant stems and wood samples are dried and milled during processing steps for chemical compositional analysis. The approach is well standardized (e.g., Sluiter et al. [34]) and provides a mean value of the sugars/lignin present in a fixed volume/weight of plant material. With this approach large quantities of plant material are frequently used during the initial phase of processing (i.e., milling) in order to give a homogeneous plant material that can be later sampled to provide statistical valid results that can be related to the original whole plant material. In addition, with *Salix*, the bark is also frequently included during the milling of stems [35]. The milling approach is, however, more difficult to apply when interests are related to specific and smaller morphological plant tissue regions. Since in the present study, we wanted to compare the chemical composition of selected stem regions (debarked and depithed) in different *Salix* clones, we applied a stem cross-sectional approach for compositional analysis. Using stem cross sections has an advantage in that analyses can be made on defined material originating from specific regions along a stem, allowing direct comparisons between stem regions and clones.

**Fig. 1:**
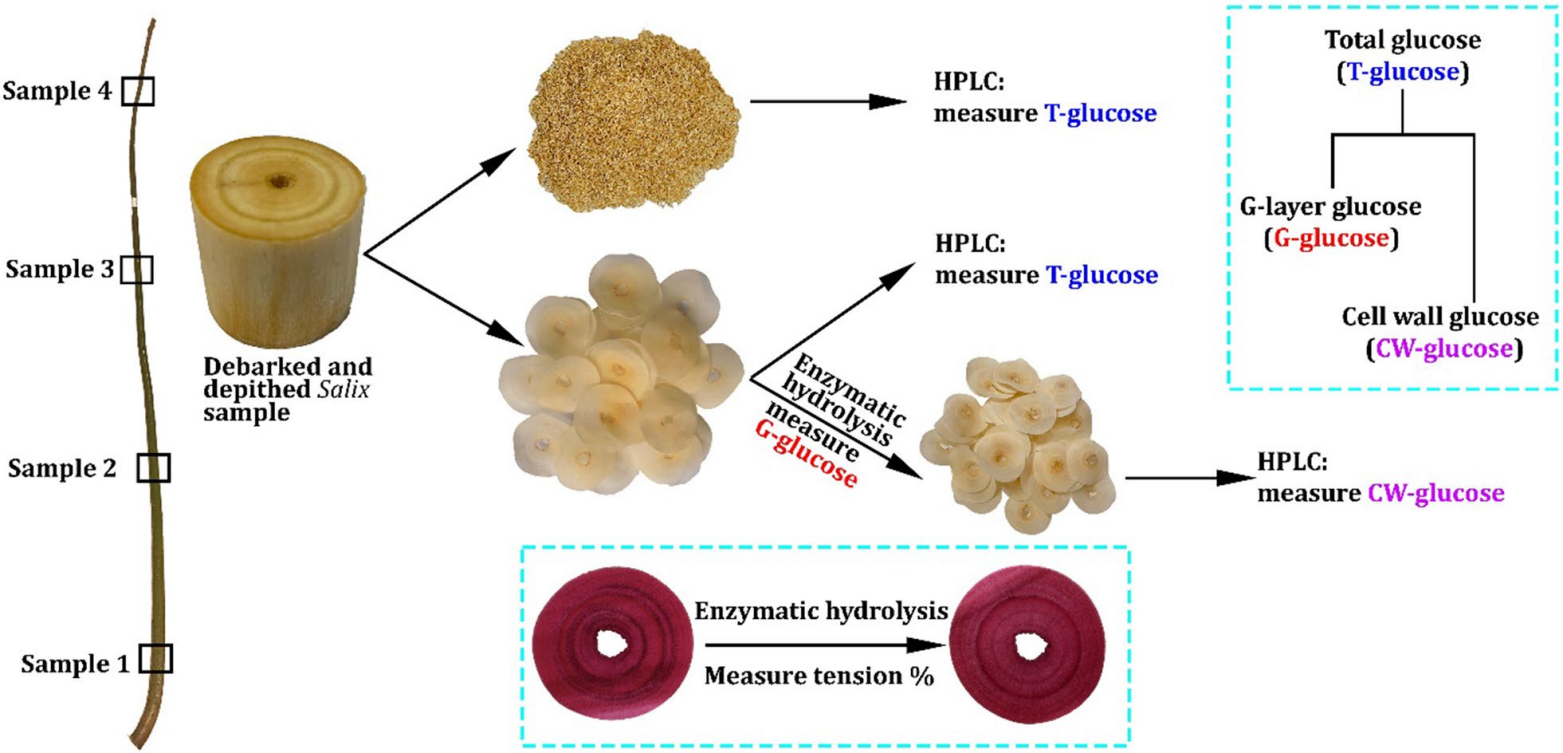
Schematic overview of the sampling and chemical/enzymatic analysis of the *Salix* stem cross sections and milled powder

**Fig. 2:**
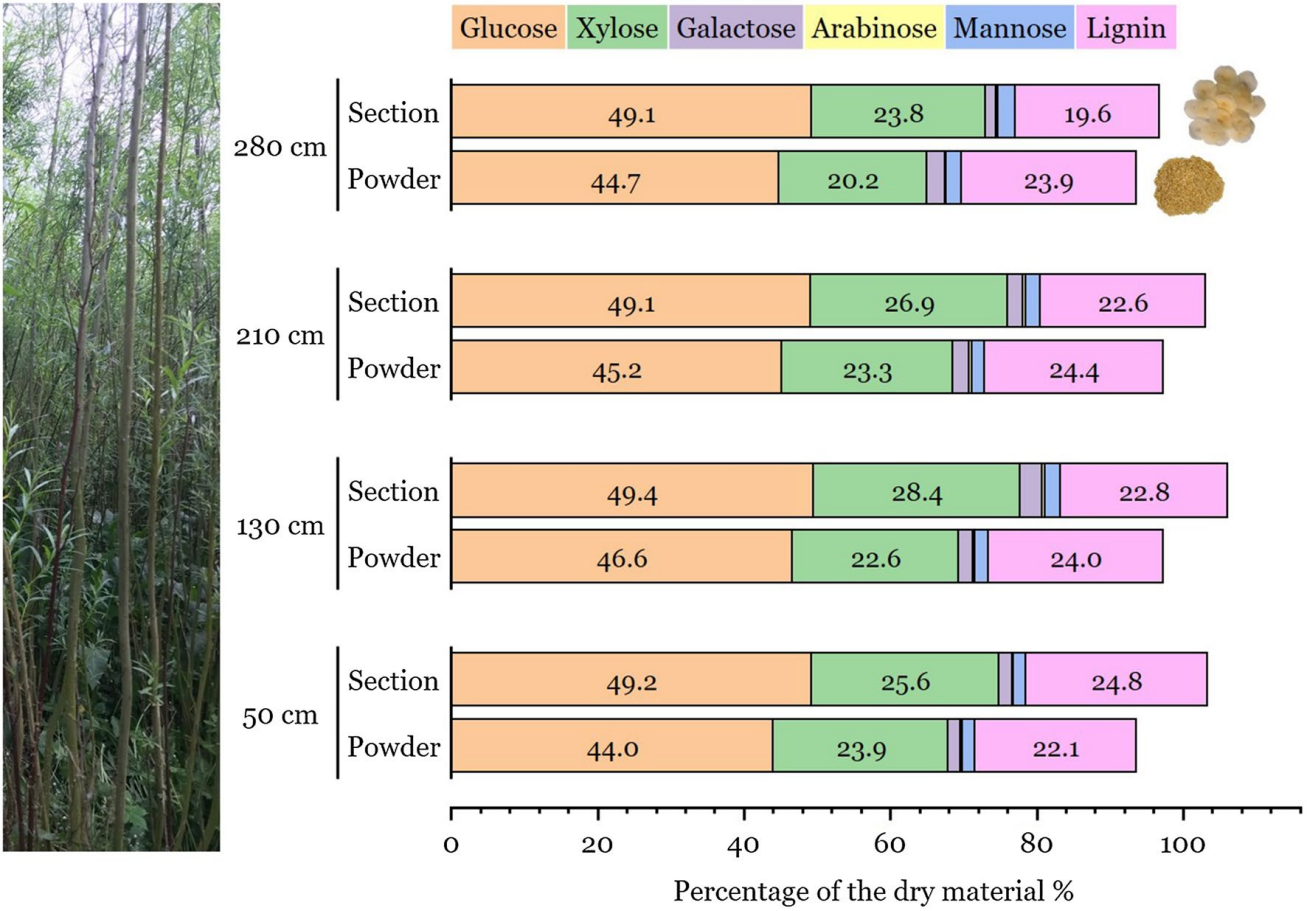
Comparison of HPLC analyses made on powder and cross sections from different stem heights of the *Salix* clone Loden

In order to test the viability of using stem sections for chemical analysis of *Salix* samples, we conducted preliminary studies on both homogenized stems regions (i.e., powder) and semi-thin sections (i.e., non-milled samples) from one clone Loden. Comparative samples were taken along the stem (Fig. 1, Table 1). To aid comparisons, the *Salix* sections and homogenized powder came from adjacent 5 cm stem regions taken at four points along the stem (Fig. 1). The percentage total glucose (T-glucose, i.e., total cell wall and tension wood glucose) determined using HPLC ranged from 49.1 to 49.4% and 44.0 to 46.6% from the analysis of the *Salix* sections and powder, respectively (Fig. 1, Table 1). The percentage total xylose ranged between 23.8 and 28.4% for sections and 20.2 and 23.9% for the powder. This represents a variation in total glucose and xylose between the sections and the powder of ca. 1.7–5.7% and 2.8–5.2%, respectively, with slightly higher glucose levels found in sections than in powder form at the different stem heights (Fig. 1, Table 1). The results are similar and well within that published for *Salix* sugar compositions [30, 36–39]. No significant differences were observed for mannose, galactose, and arabinose (*p* = 0.05, 0.9 and 0.2, respectively, according to paired t-test). The total lignin content in sections and powder varied from ca. 20–25% and 22–24%, respectively (Table 1; Fig. 2). However, the mean value for the whole Loden stem (i.e., mean of the 4 samples points) was 22.5% for sections and 23.6% for powder (Table 1). Comparing results between using sections and powder (*p* = 0.5, by one-way ANOVA) and within groups at different stem heights (according to paired t-test) gave no significant differences in the lignin values.

**Table 1:** Sugar analysis of *Salix* clone Loden using powder and serial sections from adjacent stem regions and different stem heights

*Salix*	Stem height (cm)	T-Glucose (%)	Xylose (%)	Galactose (%)	Arabinose (%)	Mannose (%)	ASL (%)	AIL (%)	Total lignin (%)
Loden sections	280	49.1	23.8	1.5	0.3	2.4	3.5	16.1	19.6
	210	49.1	26.9	2.1	0.4	2.0	3.2	19.5	22.6
	130	49.4	28.4	2.9	0.4	2.2	2.8	20.0	22.8
	50	49.2	25.6	1.8	0.2	1.7	3.1	21.6	24.8
									Mean: 22.5
Loden powder	280	44.7	20.2	2.5	0.1	2.1	2.9	21.1	23.9
	210	45.2	23.3	2.3	0.4	1.7	1.7	22.7	24.4
	130	46.6	22.6	2.0	0.3	1.8	2.9	21.1	24.0
	50	44.0	23.9	1.6	0.2	1.7	3.5	18.6	22.1
									Mean: 23.6

Composition of *Salix* expressed as % of dry raw material

The small variations in total glucose and xylose between the section and powder approaches may reflect slight differences in manner of processing, sample size, and native origin of sample. Possible explanations include as follows: (i) Slight differences in the amount of TW along the 10 cm stem cylinder used and thus differences in original tissue composition (i.e., fibers vs vessels) and thereby in native chemical composition; (ii) Effects on G-layer cellulose during milling to produce the homogenized powder. During milling of dry *Salix* wood the abrupt change in cellulose microfibril angle between the G-layer and outer S2 layer would cause the latter to be easily detach from the S2 layer which together with the known high crystallinity of G-layers [18] could cause it to break up into very small particles not retained on the sieves during the initial stages of sample preparation. The milled powder for HPLC analysis was fractionated according to the standard particle size using Retsch GmbH sieves. According to Sluiter et al. [34], only fractions passing through a 40-mesh (ca. 425 µm) and retained on a 60-mesh (ca. 250 µm) should be used for standard HPLC analysis of plant (wood) materials and other fractions excluded. Thus, the percentage of the chemical components using HPLC may be slightly over or under estimated compared to the true native wood material. In contrast, the stem cross sections were cut using a sliding microtome where section thickness is controlled within the range ca. 30–45 µm and used directly for chemical analysis. The present results and previous light microscopy studies have further shown the G-layer is retained within the G-fiber structure during microtome sectioning [30, 33].

The complete acid hydrolysis and sugar quantification process for sections were adapted according to Sluiter et al. [34] by replacing the stem powder with exactly the same amount of completely dried (103 ± 2 ℃) sections from debarked and depithed stems. Thus, by using sections instead of powder for chemical compositional analyses, the number of steps and variations during processing can be reduced and a direct morphological relationship maintained. The precision of the section method was further verified by determining the variation in both the retention times of sample peaks with standards and concentration values of each sugar component during analysis with reported values.

In summary, despite some slight variations between sections and powder along stems, the percentage of different sugars from the sections was similar to values reported in the literature [39], indicating that our modified method using sections was precise and consistent for the sugars/lignin, and thus may be considered as having potential for use as a standard method for chemical analysis of small targeted plant biomass regions.

### Estimation of tension wood in *Salix* stem cross sections using image analysis

**Fig. 3:**
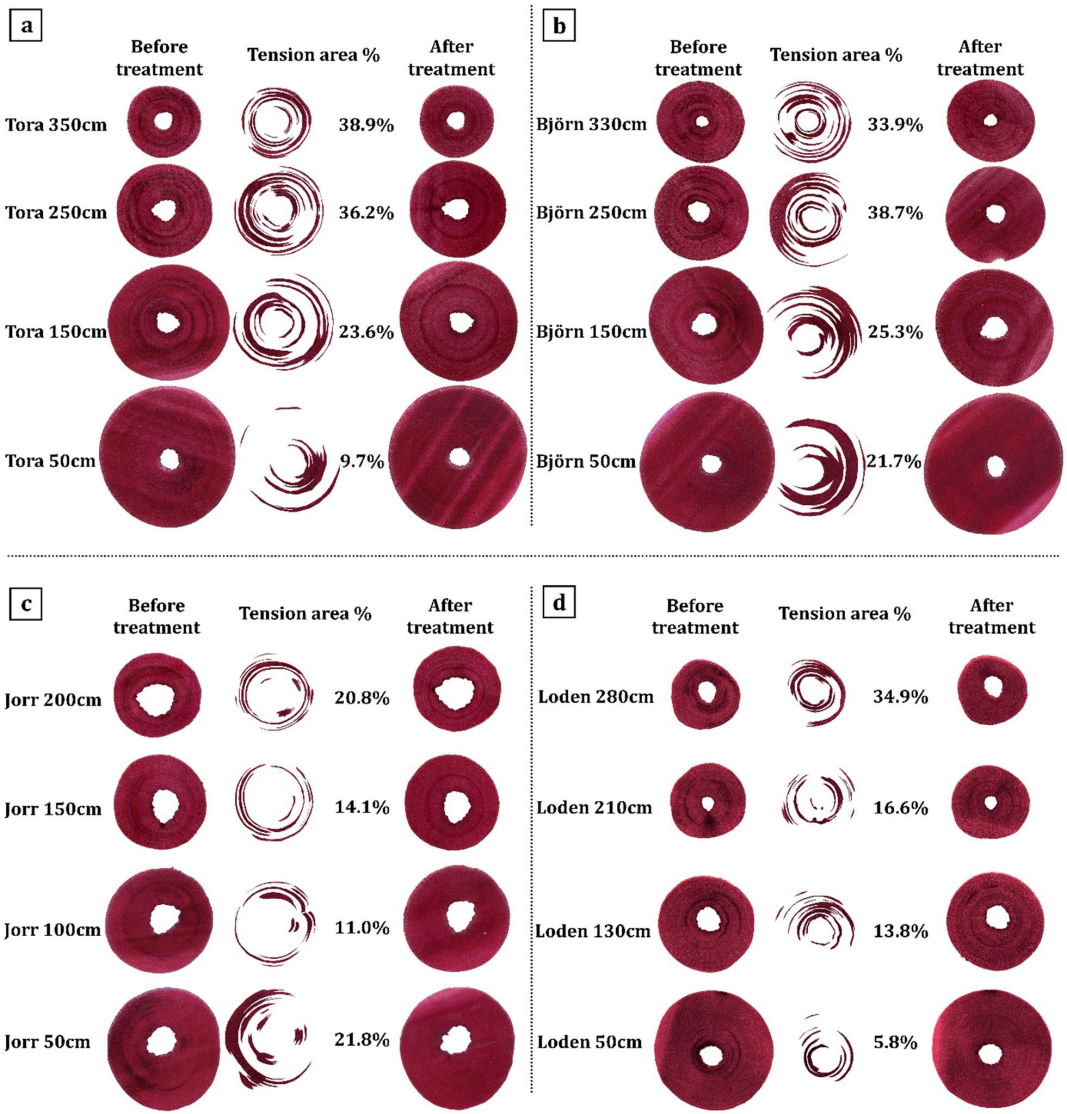
Overview of Tora (**a**), Björn (**b**), Jorr (**c**), and Loden (**d**) debarked and depithed cross sections taken from different stem heights, before and after 3-day cellulose hydrolysis (tension wood removal) and after staining with chlorazol black E and safranin. Quantification of tension wood area (as %) was done using Photoshop

Representative sections used for chemical analysis were double stained by chlorazol black E and safranin (see Materials and Methods and Fig. 3) to visualize and quantify the tension wood present in cross sections from the four *Salix* clone stems at different heights (Fig. 3). TW was visualized in cross sections as discrete bands of distinct black staining (Figs. 1, 3). The percentage TW in stem cross sections varied between both the four clones and with stem height (Fig. 3). The % tension area in Tora (Fig. 3a) ranged from ca. 10% at 50 cm to ca. 39% at 350 cm with the corresponding values in Björn (Fig. 3b) of ca. 22% (50 cm) and ca. 34% (330 cm) for similar heights (Fig. 3). Stems of Jorr (Fig. 3c) and Loden (Fig. 3d) had calculated % tension areas varying from ca. 11–22% and ca. 6–35%, respectively. These results confirm our previous results of the general trend of increasing TW with increasing stem height and also variations along the stem of the two-year-old fast growing *Salix* plants [33].

### Compositional structure of *Salix* stems and sections

**Fig. 4:**
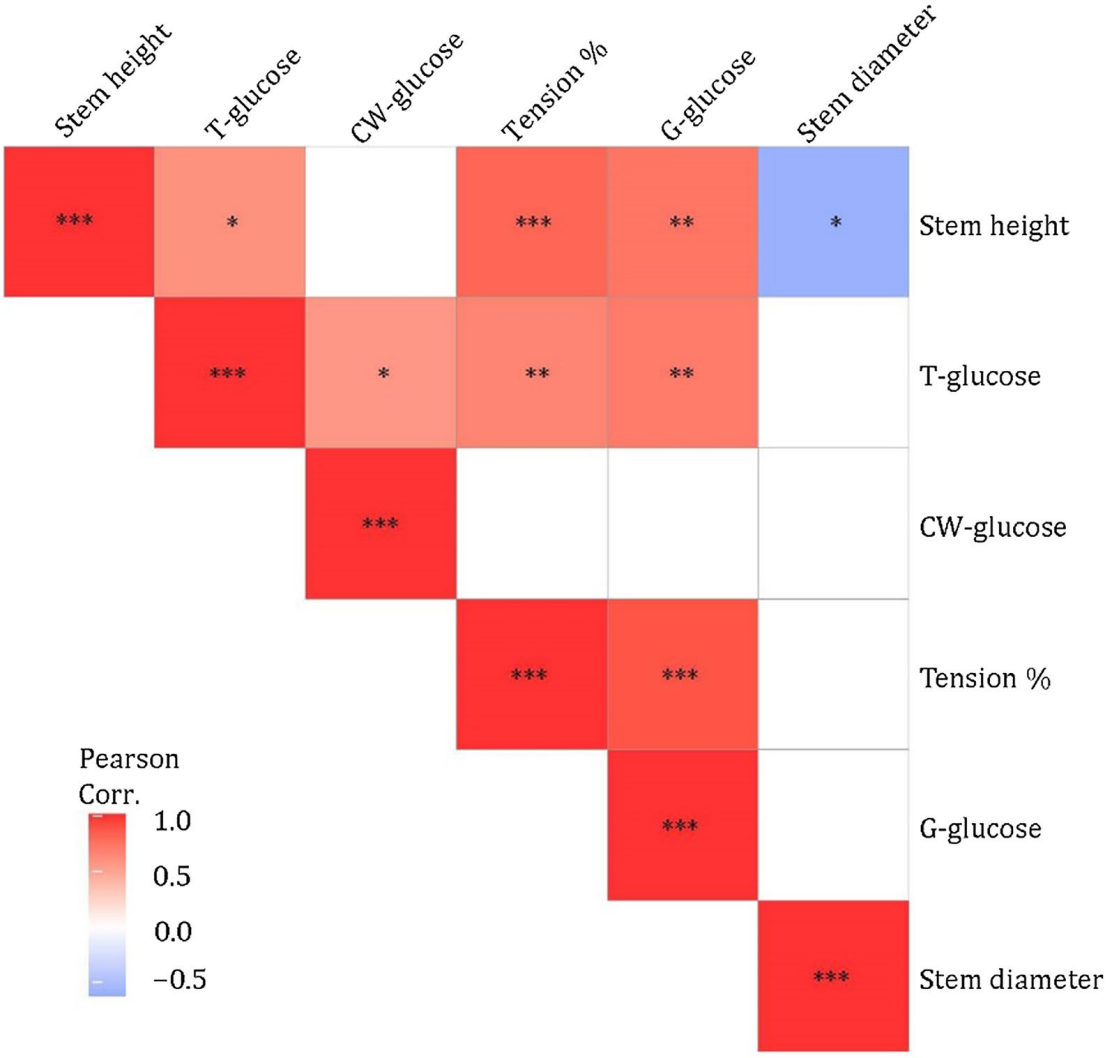
Heat map using Pearson’s correlation between six variables (Stem height, T-glucose, CW-glucose, % Tension wood, G-glucose, and stem diameter)

**Fig. 5:**
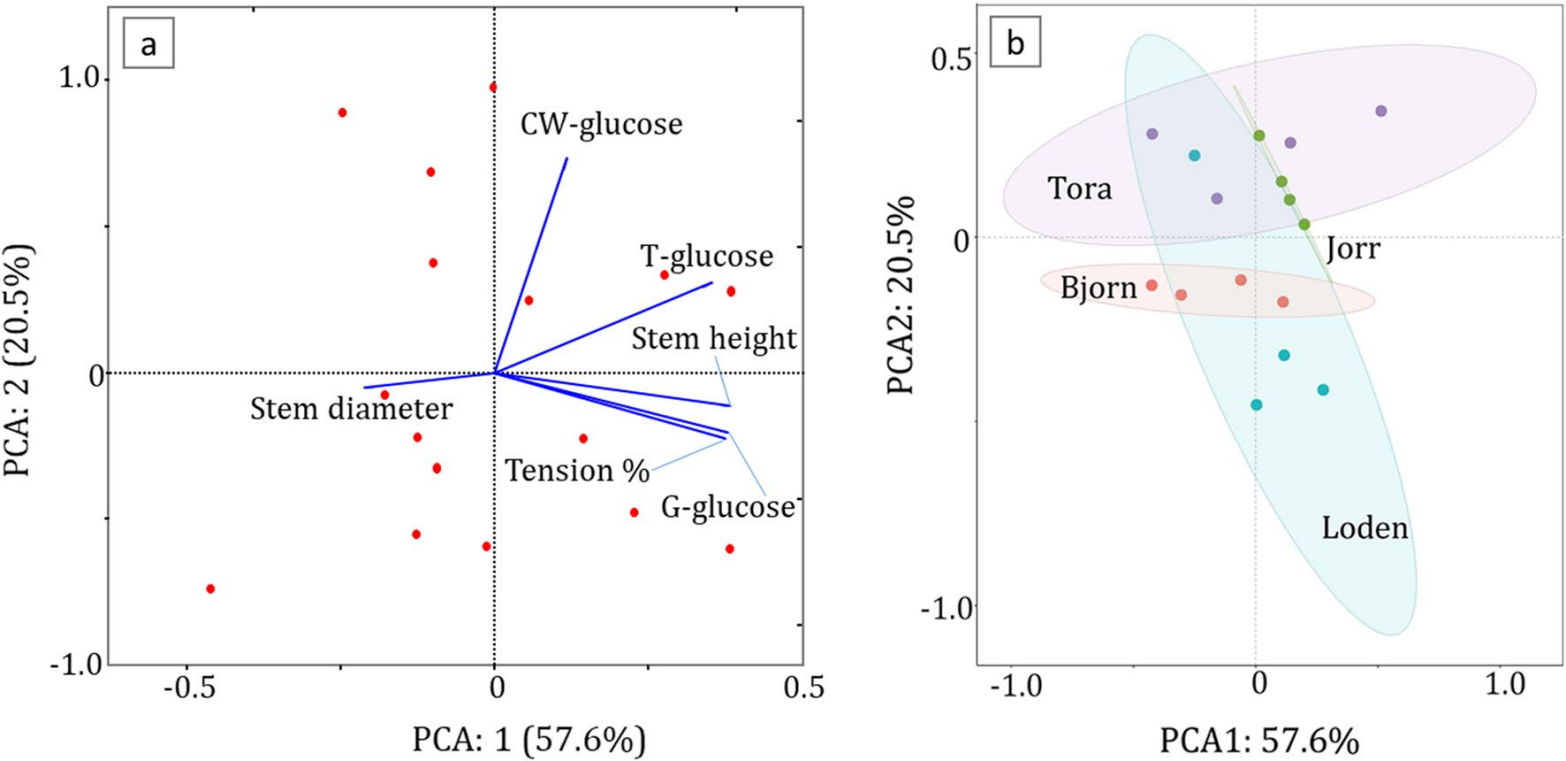
Principal component analysis (PCA) of the four *Salix* clones compared in terms of chemical and physical characteristics (i.e., T-glucose, G-glucose, CW-glucose, Tension %, Stem diameter, Stem height) of 6 elements

1. Chemical composition of *Salix* clones at different stem heights
  The chemical composition of different *Salix* spp. as biomass for biofuel production has been studied for many years, although no specific studies appear to have been conducted on the spatial chemical distribution of components along the stem. In the present work, the distribution of sugars (i.e., glucose, xylose, mannose, galactose, and arabinose) and lignin was determined for the four 2-year-old *Salix* (Tora, Björn, Jorr, and Loden) stems from top to the bottom (Table 2). All four *Salix* clones examined showed predominantly glucose (41–56%) and xylose (22–28%) as major sugars. The other sugars were relatively minor components, ranging from 0.2 to 3.9% for galactose, 0.7–2.4% for mannose and 0–0.5% for arabinose. By considering the mean value of each sugar (i.e., as % dry wt.) along the stem, the most abundant fraction was glucose with 47.7, 52.8, 49.2, and 47.1% for Tora, Björn, Loden, and Jorr, followed by xylose with 24.9, 24.1, 26.2, and 23.0%, respectively (Table 2). These values concur with most published reports on the sugar composition of *Salix* species [26, 35, 36, 39]. From a statistical aspect, the result showed some variability in glucose, both between clones (paired t-test *p* < 0.05) and at different heights (significantly different at *p* < 0.01 level, one-way ANOVA) with Tora and Björn showing increased % of glucose with stem height (results not shown). At the top of stems, Tora and Björn had 53.1 and 56.4% glucose at 350 and 330 cm stem height, respectively, which is ca. 12 and 6% higher than at 50 cm height (41.3 and 50.7%, respectively, Table 2).
2. Enzymatic saccharification of clone stem cross sections from different heights
  To estimate G-layer glucose (G-glucose) in clones at different stem heights, sections were subjected to 3-days enzymatic (cellulase) saccharification using Cellic CTec2 (See Material and methods). After saccharification, sections were randomly selected, double stained with chlorazol black E and safranin, and examined using light microscopy. As shown in Fig. 3a–d, all the darkly colored tension wood bands were removed during saccharification verifying the complete removal of the G-layer from the tension wood fibers. G-glucose released due to enzyme saccharification varied along the stems. Results showed an increase in D-glucose release with stem height, with the highest and lowest G-glucose release from the cross sections for all clones recorded at the top (i.e., 14.7% for Tora at 350 cm; 13.4% for Björn at 330 cm; 9.4%, Jorr for 200 cm; and 11.8% for Loden at 280 cm) and bottom (3.7% for Tora at 50 cm; 9.5% for Björn at 50 cm; 7.6% for Jorr at 100 cm; and 4.0% for Loden at 50 cm) of the stems, respectively, apart from clone Jorr where the lowest d-glucose level was recorded at 100 cm stem height (Table 4). Additional analysis of d-glucose release from an 8-year-old field *Salix* sample with mostly tension wood was 11.9% (Table 4).
  In agreement with our previous studies [30, 33], after enzymatic hydrolysis of stem sections, we obtained values for G-glucose ranging from 3.7 to 14.7% of the original raw material weight (as dry wt.), with the top of the two-year-old *Salix* stems generally showing higher yields of G-glucose than bottom regions. The more TW present, the more G-glucose, further proving that G-glucose is significantly and positively related to the percentage of TW (Fig. 4, Table 5).
  The total volume of TW was also determined for each clone (Table 4). Tora and Björn clones showed greater amounts of TW (24.2 and 28.2%, respectively) in the test material (debarked and depithed stem volume: ca. 993 and 968 cm^3^ for Tora at 50–350 cm and Björn at 50–330 cm, respectively), which was almost two times greater than Jorr (14.9%; stem volume at 50–200 cm is ca. 296 cm^3^) and Loden (15.5%; stem volume at 50–280 cm is ca. 364 cm^3^) (Table 4). If we consider larger *Salix* wood volumes, for example, 1 m^3^ of Tora (i.e., ca. 1007 stems at a height 50–350 cm) and Björn (i.e., ca. 1033 stems at a height of 50–330 cm), the stems would theoretically contain ca. 0.24 and 0. 28 m^3^ of TW. For the corresponding volume of 1 m^3^, 3383 stems of Jorr (50–200 cm) and 2750 stems from Loden (50–80 cm) would be required, providing 0.16 and 0.14 m^3^ of TW, respectively. This emphasizes the large differences that can be shown in TW development by the four clones studied when the biomass quantity is scaled up. It is also emphasizes the significant role likely played by the presence of TW to the glucose content.
3. Chemical composition (HPLC) of enzymatic hydrolyzed stem sections from clones at four stem heights
  In order to verify the differences and methodology, the same stem serial cross sections after enzymatic hydrolysis were re-examined using HPLC for chemical composition. Following enzymatic hydrolysis, the glucose (i.e., cell wall structural glucose = CW-glucose) content was reduced (i.e., 36.9–51.2%) as expected while the xylose content increased from 22.0–28.4% to 23.7–32.5% (Tables 2–4). Assuming the enzyme saccharification process only removed G-glucose from the G-fibers, the calculation for xylose would be % xylose (before enzyme saccharification) = xylose content/dry wt. of raw material × 100%, and % xylose (theoretical, after enzyme saccharification) = xylose content/(dry wt. of raw material – G-glucose) × 100%. Therefore, the increase in xylose content after enzyme saccharification is likely due to the loss of G-glucose material during the treatment (i.e., as a % of dry material; Table 4). The percentage glucose and xylose showed a similar trend in the 8-year-old field *Salix* sample as the two-year-old tested *Salix* sample (Table 4). The glucose reduced from 48 to 41.6%, while the xylose content slightly increased by 1.7% (Tables 2, 3).
  The calculated theoretical value of CW structural glucose and the measured result from HPLC analysis of the different clones at different stem heights were very similar (Tora, Björn, Jorr, and Loden show *p* = 0.3, 0.1, 0.5, and 1.0, respectively, according to a paired t-test), emphasizing the validity of the method (Table 4). Thus, the correlation between T-glucose, G-glucose, and CW-glucose conforms well to the expected: Total glucose = G-layer glucose + Cell wall glucose.
4. Correlation before and after enzymatic hydrolysis
  In order to determine the likely contribution played by TW-glucose to total *Salix* chemical composition and its potential for biofuels, Pearson’s correlation analysis was used to analyze the significant correlation levels among all related factors (Figs. 4, 5; Table 5). A strong and positive association was observed between T-glucose and G-glucose, % tension wood, and stem height (Pearson’s correlation coefficient value (Pearson Corr.) *r* = 0.71, 0.66 and 0.60, respectively, *p* < 0.01) (Fig. 4, Table 5). As expected, G-glucose also showed a strong and positive correlation with % tension wood and stem height (Pearson Corr. *r* = 0.89 and 0.74, respectively, *p* < 0.01), indicating that the amount of freely available glucose from the G-layer is significantly related to % tension wood. The CW-glucose along the stem (from top to bottom) only varied slightly, indicating that structural CW-glucose has no large variations (Fig. 4, Table 5). This confirms that as the TW increases, more G-glucose is available and more T-glucose detected. In this way, it should be possible to give an average value for CW-glucose that may be used as a clone related value (i.e., reference) for HPLC determined glucose that could be used as a standard to compare not only different clones but also the same and different clones from different locations. Higher values will indicate not only greater levels of TW and glucose but also more readily available glucose for hydrolysis.
  Principal component analysis (PCA) was also used to understand the scale of impact of chemical component parameters and physical measurement on individual *Salix* stems. For analysis, CW-glucose, T-glucose, G-glucose, Tension %, Stem height, and Stem diameter were used as variables. Figure 5a shows the graphical representation of the PCA results where 6 properties are used per observation. The first component PCA1 explains 57.6% of the variation, and the second component PCA2 20.5%. The influence of variables on the observation is indicated by their length, direction, and angle with the longer length of the variable indicative of higher influence on conservation and shorter length less influence. Figure 5b displays the relationship between all samples from different stems and different heights. The two principal components’ relation between different stems showed that Tora and Björn are more similar as are Jorr and Loden with respect to physical and chemical analyses used (Fig. 5b).
5. Comparison of Total glucose and G-glucose in different clones
  Comparison of total glucose (T-glucose) as a % of starting raw material gave values along the clone stems of ca. 41.3–53.1% (mean 47.7%) for Tora, ca. 50.7–56.4% (mean 52.8%) for Björn, ca. 45.6–49.1% (mean 47.1%) for Jorr, and ca. 49.1–49.4% (mean 49.2%) for Loden (Table 4). Comparative values for G-glucose released by enzyme saccharification were ca. 3.7–14.7% (mean 8.9%) for Tora, ca. 9.5–13.4% (mean 11.5%) for Björn, ca. 7.6–9.4% for Jorr (mean 8.6%), and ca. 4.0–11.8% (mean 6.6%) for Loden (Table 4). As a comparison, this indicates a contribution of between ca. 6.6 and 11.5% G-glucose to the total glucose with Björn > Tora > Jorr > Loden for the clone test material. These values for G-glucose are slightly lower than that determined previously [33], but nonetheless indicates a significant contribution of G-glucose from the presence of TW fibers with gelatinous layers. Previously we calculated the possible effect of G-glucose based on a literature mean value for stem glucose (i.e., cellulose) of 45% [33] without any prior knowledge of the chemical composition of stems. Here, however, we make comparisons based on analyses of the same *Salix* stem materials. It is highly probable that the wide variations reported in the literature of total glucose (cellulose) actually also include a percentage TW and that the variation in actual cell wall structural glucose (cellulose not in the G-layer) is much less. However, by using wet chemical analysis approaches and milling of the entire wood material, it is not possible to distinguish between them.
6. Determination of lignin in *Salix* clones at different stem heights both before and after enzymatic saccharification.
  Stems of the four *Salix* clones showed a total lignin content [i.e., acid-soluble lignin (ASL) + acid-insoluble lignin (AIL)] ranging from ca. 18 to 25% at different stem heights (Table 6) which is consistent with published results. Serapiglia et al. [36], for example, reported a 19–22% lignin content for 25 two-year-old debarked willow shrub clones and Dou et al., 22.4% lignin in a debarked 4-year-old willow hybrid “Karin” [14].
  Before enzyme saccharification, in 3 (i.e., Tora, Björn, Jorr) of the 4 clones, the highest AIL was recorded in the upper parts of the stem in contrast with Loden that showed the opposite result (Table 6). Using Pearson’s correlation analysis, the ASL showed moderate and negative correlation with stem height, T-glucose, and % tension wood (i.e., − 0.65, − 0.64, and − 0.44, 0.01 < *p* < 0.1) (data not shown in Table 6). Since the percentage of total ASL contributed less than ca. 5% of the total lignin content, the ASL should not significantly affect the compositional results for lignin. By using the mean lignin content of the whole stem (i.e., mean of the four analysis points along the clone stems), we can compare the total mean lignin content of the whole stem which was very similar with a difference less than 0.4% for all clones (Table 6). This is similar to the results of Cui et al. [39] who studied 3-year-old *Salix integra* and found no significant differences in lignin distribution along the stem. At 10 cm above ground they found 21.3% AIL and 4.9% ASL, at 120 cm 20.0% AIL and 4.3% ASL and at 210 cm 19.3% AIL and 4*.*3% ASL, respectively. Results suggest therefore that in *Salix* biomass, the percentage of TW will not be a significant factor affecting total lignin.
  Some slight variation in lignin for the clones was noted for sections before and after enzyme saccharification with mean stem values ranging from 22.4 to 22.8% before and 22.4 to 24.3% after the main difference noted for clones Tora (1.5%) and Loden (1.4%). Currently, the reason for the slight increase is unknown as all studies were conducted on debarked and depithed stems. Both the ASL and AIL contributed to the slight increase.

**Table 2:** Sugar (HPLC) analysis of sections from four *Salix* clones taken at four stem heights made before enzyme treatment

*Salix*	Stem height (cm)	Glucose (%)	Xylose (%)	Galactose (%)	Arabinose (%)	Mannose (%)
Tora	350	53.1	24.2	3.7	0.5	2.2
	250	49.9	25.3	2.9	0.4	2.1
	150	46.6	24.7	2.4	0.4	1.8
	50	41.3	25.5	2.2	0.3	1.4
	Mean	47.7	24.9	2.8	0.4	1.9
Björn	330	56.4	26.1	3.9	0.4	2.2
	250	52.5	23.7	3.3	0.4	2.1
	150	51.7	24.2	3.0	0.3	1.7
	50	50.7	24.0	2.7	0.3	1.6
	Mean	52.8	24.1	3.2	0.4	1.9
Jorr	200	47.1	22.0	1.7	0.3	2.3
	150	45.6	23.0	0.8	0.0	2.1
	100	46.5	23.4	0.2	0.0	1.5
	50	49.1	23.6	1.4	0.0	2.2
	Mean	47.1	23.0	1.0	0.1	2.0
Loden	280	49.1	23.8	1.5	0.3	2.4
	210	49.1	26.9	2.1	0.4	2.0
	130	49.4	28.4	2.9	0.4	2.2
	50	49.2	25.6	1.8	0.2	1.7
	Mean	49.2	26.2	2.1	0.3	2.1
Field *Salix*		48.0	26.6	1.6	0.0	0.7

**Table 3:** Sugar (HPLC) analysis of sections from four *Salix* clones taken at four stem heights made after enzyme treatment

*Salix*	Stem height (cm)	Glucose (%)	Xylose (%)	Galactose (%)	Arabinose (%)	Mannose (%)
Tora-E	350	45.3	31.7	2.4	0.1	1.6
	250	44.9	29.8	2.5	0.2	1.6
	150	40.6	25.0	2.8	0.4	1.5
	50	36.9	23.7	2.2	0.4	1.2
	Mean	41.9	27.5	2.5	0.3	1.5
Björn-E	330	49.8	32.5	3.2	0.3	1.4
	250	51.2	31.0	3.4	0.3	1.5
	150	47.8	29.8	3.7	0.4	1.5
	50	47.5	31.3	3.5	0.4	1.7
	Mean	49.1	31.1	3.5	0.4	1.5
Jorr-E	200	41.1	24.1	1.1	0.0	2.0
	150	42.8	24.3	1.1	0.0	1.9
	100	42.8	24.9	0.7	0.0	1.7
	50	41.7	25.4	0.0	0.0	1.5
	Mean	42.1	24.7	0.7	0	1.8
Loden-E	280	44.7	29.2	2.7	0.2	2.1
	210	49.1	26.9	2.1	0.4	2.0
	130	46.1	27.6	2.1	0.2	2.1
	50	45.9	27.1	1.8	0.2	2.1
	Mean	46.5	27.7	2.2	0.3	2.1
Field *Salix*-E		41.6	28.3	1.6	0.0	0.4

**Table 4:** Comparison of total glucose (T-glucose) using HPLC with G-glucose from enzymatic hydrolysis (as % of starting raw material) for four *Salix* clones at different stem heights together with % tension wood at different stem heights

*Salix*	Stem height (cm)	T-glucose	G-glucose	Theoretical CW-glucose	Theoretical CW-glucose	HPLC measured CW-glucose	Tension area (%)	Stem diameter (cm)	Tension volume (%)
		% raw material			% enzymatic hydrolyzed material				
Tora	350	53.1	14.7	38.4	45.1	45.3	38.9	1.4	24.2
	250	49.9	9.6	40.3	44.5	44.9	36.2	1.8	
	150	46.6	7.5	39.1	42.3	40.6	23.6	2.2	
	50	41.3	3.7	37.6	39.0	36.9	9.7	2.6	
	Mean	47.7	8.9	38.9	42.7	41.9			
Björn	330	56.4	13.4	43.0	49.6	49.8	33.9	1.6	28.2
	250	52.5	12.4	40.1	45.7	51.2	38.7	1.8	
	150	51.7	10.8	40.9	45.8	47.8	25.3	2.2	
	50	50.7	9.5	41.2	45.5	47.5	21.7	2.7	
	Mean	52.8	11.5	41.3	46.7	49.1			
Jorr	200	47.1	9.4	37.7	41.7	41.1	20.8	1.4	15.5
	150	45.6	8.0	37.6	40.9	42.8	14.1	1.5	
	100	46.5	7.6	38.9	42.1	42.8	11.0	1.7	
	50	49.1	9.2	39.9	43.9	41.7	21.8	1.8	
	Mean	47.1	8.6	38.5	42.2	42.1			
Loden	280	49.1	11.8	37.3	42.3	44.7	34.9	1.1	14.0
	210	49.1	5.3	43.8	46.2	49.1	16.6	1.2	
	130	49.4	5.1	44.3	46.7	46.1	13.8	1.5	
	50	49.2	4.0	45.2	47.1	45.9	5.8	1.9	
	Mean	49.2	6.6	42.7	45.6	46.5			
Field *Salix*		48.0	11.9	36.1	41.0	41.6			

T-glucose: total glucose; G-glucose: tension wood glucose; CW-glucose: cell wall structural glucose

**Table 5:** Pearson’s correlation analysis for correlation between T-glucose, G-glucose, CW-glucose, % Tension wood, Stem diameter, and Stem height

		T-glucose	G-glucose	CW-glucose	% Tension wood	Stem diameter	Stem height
T-glucose	Pearson Corr.	1	0.71	0.57	0.66	− 0.24	0.60
	*p*-value	–	0.0019	0.020	0.0050	0.37	0.01
G-glucose	Pearson Corr.		1	− 0.024	0.89	− 0.25	0.74
	*p*-value		–	0.93	0.0000041	0.35	0.001
CW-glucose	Pearson Corr.			1	0.040	– 0.15	0.12
	*p*-value			–	0.88	0.57	0.65
% Tension wood	Pearson Corr.				1	– 0.24	0.81
	*p*-value				–	0.37	0.00015
Stem diameter	Pearson Corr.					1	– 0.61
	*p*-value					–	0.01
Stem height	Pearson Corr.						1
	*p*-value						–

Two-tailed test of significance used

**Table 6:** Lignin analysis of sections from four *Salix* clones taken at four stem heights made before and after enzyme saccharification

*Salix*	Stem height (cm)	Before enzyme saccharification				After enzyme saccharification			
		ASL (%)	AIL (%)	Total (%)	Mean lignin of the entire stem (%)	ASL (%)	AIL (%)	Total (%)	Mean lignin of the entire stem (%)
Tora	350	2.4	22.2	24.6	22.8	4.2	23.0	27.2	24.3
	250	3.2	20.6	23.8		3.9	21.3	25.2	
	150	3.6	17.2	20.8		3.7	19.8	23.4	
	50	4.0	18.0	22.1		4.3	17.0	21.3	
Björn	330	2.8	20.4	23.2	22.4	4.1	19.7	23.8	22.8
	250	3.2	18.5	21.7		3.7	20.5	24.2	
	150	3.6	18.5	22.1		3.6	18.8	22.4	
	50	3.8	18.9	22.7		3.5	17.5	21.0	
Jorr	200	3.9	20.1	23.9	22.5	3.7	20.4	24.2	22.4
	150	3.9	20.3	24.2		4.3	18.2	22.6	
	100	4.3	19.5	23.8		4.8	16.2	21.0	
	50	4.4	13.6	18.0		4.5	17.2	21.7	
Loden	280	3.5	16.1	19.6	22.4	3.7	20.9	24.6	23.8
	210	3.2	19.5	22.6		3.6	19.8	23.4	
	130	2.8	20.0	22.8		3.6	18.4	22.0	
	50	3.1	21.6	24.8		3.5	21.9	25.4	
Field *Salix*		1.9	17.6	19.5		2.2	21.2	23.4	

## Conclusions

The methodology of using stem serial sections directly instead of/or to complement milled powder for chemical (HPLC) analyses represents a viable approach. Comparisons of the percentage of different sugars measured along stems at four heights in four *Salix* clones were comparable to literature values. In particular, the use of cross sections has the advantage in allowing chemical analyses of small biomass quantities from targeted tissue plant regions.

Using a combination of histochemical/microscopy, image, enzymatic, and chemical analyses of stem sections and milled powder, the presence and importance of tension wood (TW) in the stems of four *Salix* clones at four heights were characterized. Analyses not only provided details on the contribution played by total / individual sugars and lignin in the stems examined but also allowed the contributions of total glucose (T-glucose) to be divided into that derived from structural cell wall glucose (CW-glucose) and that from tension wood (TW) glucose (G-glucose).

Tension wood was present in all 2-year-old *Salix* stems at the four stem heights examined, with % TW increasing with stem height. Percentage G-glucose from enzymatic saccharification was found significantly and positively correlated with % tension wood in stems. The mean % G-glucose and % tension volume for the entire stem were Björn > Tora > Jorr > Loden.

Analysis of the total sugar and lignin content of the four *Salix* clones before and after enzyme saccharification showed CW-glucose and lignin as not significantly affected by the presence of TW or stem height, but rather the *Salix* clone. Determined and theoretical values showed good correlation of Total Glucose = CW-glucose + G-glucose. Thus, the presence of TW and particularly G-fiber gelatinous layers (G-glucose) can significantly increase the potential of readily available d-glucose for biofuel production.

Combining the stem sectional approach with chemical analysis (i.e., HPLC/enzymatic saccharification) has potential as a method for selecting *Salix* clones from *on-going* field experiments. If a reference value of cell wall structural glucose can be determined (e.g., ca. 38–43% glucose of dry wt. raw material, for the two-year-old *Salix* clones used in the current study), or is known, higher values will represent the contribution of G-layer glucose; with the higher the value the more significant the contribution of a “lignin-free” glucose source for biofuel production.

## Materials and methods

### Plant materials and harvesting

The two-year-old *Salix* stems used for studies were derived from a short rotation plantation experiment on arable land in Uppsala, Central Sweden (59° 49′ N, 17° 39′ E) [40], September 2020. The varieties were taxonomically distinct at species or genotype level and included two full siblings “Björn” (*Salix schwerinii* E. Wolf. × *S. viminalis* L.) and “Tora” (*S. schwerinii* × *S. viminalis*) as well as “Jorr” (*S*. *viminalis*) and “Loden” (*S. dasyclados* Wimm.), the latter most distinct in terms of taxonomy from the other three varieties [40]. The stems were randomly selected from a large-scaled experimental block design, cut ca. 10 cm above the soil surface and transported to the laboratory, and maintained at − 20 ℃ until further use [33, 40]. In previous studies, these *Salix* clones particularly Tora and Björn were shown as excellent cultivars for producing large biomass quantities [22].

### Experimental design (Fig. 1)

All physical measurements (i.e., stem diameter) and chemical analyses were made on debarked and depithed samples. Ten cm stem cylinders were sawn at four-point intervals starting at 50 cm height of the stem (Fig. 1). The *Salix* cylinders were divided into two parts: (i) the upper part (ca. 5 cm) was dried (103 ± 2 ℃) overnight and milled (see below) into powder for chemical analyses (HPLC), and (ii) the lower part of the stem was serial sectioned for subsequent chemical analyses, enzyme saccharification, and determination of % area TW. In step one, chemical analyses were conducted to measure the total sugar (i.e., total glucose, T-Glucose) and lignin present in adjacent stem areas at four points along the stem for one clone (i.e., Loden). Enzyme saccharification was performed to determine the freely available glucose (G-glucose), derived from the TW G-layers. Following enzymatic saccharification, the same *Salix* sections were used for subsequent chemical (HPLC) analysis to determine the residual structural cell wall glucose (CW-glucose) present. This approach allowed direct comparison of total stem glucose from a closely adjacent stem region after milling, with the glucose derived from sections after enzyme saccharification and with the total glucose remaining in the same sections. The approach allowed therefore comparison of the use of serial stem cross sections vs powder from homogenized stem regions (Fig. 1), as well as comparisons of chemical compositions in discrete tissue regions.

### Chemical analyses using HPLC

Preliminary investigations were conducted on both *Salix* powder and stem sections from clone Loden in order to standardize the approach for chemical analyses. Using 10 cm stem cylinders, the upper part (5 cm) after drying (103 ± 2 ℃) was milled using a Retsch SM100 mill to pass a 40-mesh screen with materials retained on a 60-mesh screen of size 250–425 µm. The lower *Salix* cylinder (i.e., 5 cm) was cut into serial cross section using a sliding microtome (Leica 1300 sledge microtome) at a nominal section thickness of ca. 30–45 µm and processed directly without milling. It was found that the serial sections were easily dissolved in the standard preparative process for sugars and lignin described below. Both the powders and serial sections were separately analyzed for ASL, AIL, and monosaccharides, according to Sluiter et al. [34]. ASL was determined using a Hitachi U-2910 spectrophotometer (Hitachi, Tokyo, Japan) with an absorptivity of 110 L/g/cm at a wavelength of 205 nm. Monomeric carbohydrates were determined using a Chromaster high-performance chromatography (HPLC; Hitachi, Tokyo, Japan) system equipped with an evaporative light scattering detector (ELSD-90; VWR International GmbH, Darmstadt, Germany), and a Metacarb 87P column (300 mm × 6.5 mm; Santa Clara, CA, USA) with a guard column (Metacarb 87P 50 mm × 4.6 mm). ELSD-90 was operated at 50 °C, 2.5 bars, and N_2_ was used as the nebulizing gas. The sugars were eluted using ultrapure water as a mobile phase at a constant flow rate of 0.5 mL/min and column temperature of 85 °C. Monosaccharides (i.e., glucose, xylose, galactose, mannose, arabinose) used for standards were purchased from Sigma-Aldrich Co. Water used throughout the experiments was purified on a Milli-Q system. All other chemicals and solvents used were of analytical grade for HPLC.

### Sectioning and staining of stem cross sections for tension wood analysis

Serial cross sections were stained with 1% w/v chlorazol black E+0.1% w/v safranin [33] to visualize presence of TW. Chlorazol black E stains the gelatinous layer (G-layer) of TW fibers strongly black, the double staining giving optimal staining for image analysis. Stains were purchased from Sigma-Aldrich (St. Louis, USA). Entire stem cross sections were stained and scanned using an Epson Perfection Pro 750 film scanner with a pixel resolution of 2400 dpi and areas of TW marked as previously described [33]. Thereafter, using Adobe Photoshop CC 2017, the pixel values of the selected TW area and the whole section area can be determined. The % area of TW in entire stem cross sections was then quantified using the equation: area of TW in pixels / area of the whole section in pixels × 100%. From this, the total volume in kg/m^3^ for individual sections of the stem could be calculated.

### Enzyme saccharification

Conditions for enzymatic saccharification of the *Salix* stem sections were as previously reported [33]. According to the manufacturers, the enzyme “blend” contains a cocktail of cellulases (Cellic CTec2) and is recommended for industrial treatment of lignocellulose biomass [41]. Our previous studies involved time period, enzyme concentration, number of *Salix* sections sufficient for quantification of d-glucose production, and studies on the effect of pre-drying sections at 103 ± 2 ℃ for dry weight determination before enzyme treatment as well as observations on the progressive removal of the G-layer using light microscopy after staining with chlorazol black E and safranin. Detection of free d-glucose was done using the glucose oxidase/peroxidase (GOPOD) assay kit according to the manufacturer’s instructions (Megazyme, Bray, Ireland). Stem cross sections were incubated in 150 mL Erlenmeyer flasks containing 100 µL Cellic CTec2 enzyme in 50 mL citrate buffer (pH 5) at 50 ℃ and at 100 rpm (i.e., sufficient to keep sections slightly moving and separate from each other) for 3 days in an INNOVA 4000 rotary incubator (Instrument AB, Lambda, Sweden). One hundred microliters of 95% ethanol was used as bactericide. A minimum of 12 serial stem cross sections (i.e., ca. 350–400 mg wood, dry wt.) per flask was used depending on stem cross-section area. For sections from the upper regions of stems (i.e., 250–350 cm) where the diameter of the stem was small, greater numbers of sections were required for quantification of d-glucose.

### Calculations and statistical analyses

Three determinations of glucose were made: (i) Total stem glucose (G-glucose) determined using HPLC, (ii) G-glucose (G-glucose) determined after enzymatic saccharification of stem sections, and (iii) Cell wall (structural) glucose (CW-glucose) determined on stem sections using HPLC following enzymatic saccharification and removal of G-glucose. All chemical and enzymatic analyses were performed in duplicate (often in triplicate) samples and 3 technical replicate analyses were made for each sample. The sugar concentrations were expressed in grams per 100 g dry wt. raw material.

To estimate the cell wall structural glucose (CW-glucose), the theoretical value of CW-glucose of enzymatic hydrolyzed samples was calculated by the following equation:

$${\text{Theoretical value of CW-glucose}} (\text{i.e. }\% \text{enzymatic hydrolyzed material}) = (\text{T-glucose} - \text{G-glucose})/(100 - \text{G-glucose}) \times 100 \%,$$

T-glucose: percentage of T-glucose from HPLC analysis (as % of raw material);

G-glucose: percentage of G-glucose released during the enzymatic hydrolysis process (as % of raw material).

Statistical calculations and principal component analysis (PCA) were conducted using Origin 2020 software (OriginLab, Serial Number: GF3S5–3089–7904574). The one-way ANOVA and paired samples t-test (two tailed) was applied to analyze significant differences. Pearson correlation coefficient was used with the two-tailed test of significance. PCA was analyzed using standardize methods and confidence interval of 90%.

## Data Availability

The data and material used to support the findings of this study are included within the article.
